# *Bacillus pumilus* Group Comparative Genomics: Toward Pangenome Features, Diversity, and Marine Environmental Adaptation

**DOI:** 10.3389/fmicb.2021.571212

**Published:** 2021-05-07

**Authors:** Xiaoteng Fu, Linfeng Gong, Yang Liu, Qiliang Lai, Guangyu Li, Zongze Shao

**Affiliations:** ^1^Key Laboratory of Marine Genetic Resources, Third Institute of Oceanography, Ministry of Natural Resources, Xiamen, China; ^2^State Key Laboratory Breeding Base of Marine Genetic Resources, Xiamen, China; ^3^Key Laboratory of Marine Genetic Resources of Fujian Province, Xiamen, China; ^4^Southern Marine Science and Engineering Guangdong Laboratory, Zhuhai, China; ^5^State Key Laboratory of Applied Microbiology Southern China, Guangdong Provincial Key Laboratory of Microbial Culture Collection and Application, Guangdong Open Laboratory of Applied Microbiology, Guangdong Microbial Culture Collection Center (GDMCC), Guangdong Institute of Microbiology, Guangdong Academy of Sciences, Guangzhou, China

**Keywords:** *Bacillus pumilus* group, phylogenetics, pan-genome, marine adaptation, differentiation

## Abstract

**Background:**

Members of the *Bacillus pumilus* group (abbreviated as the *Bp* group) are quite diverse and ubiquitous in marine environments, but little is known about correlation with their terrestrial counterparts. In this study, 16 marine strains that we had isolated before were sequenced and comparative genome analyses were performed with a total of 52 *Bp* group strains. The analyses included 20 marine isolates (which included the 16 new strains) and 32 terrestrial isolates, and their evolutionary relationships, differentiation, and environmental adaptation.

**Results:**

Phylogenomic analysis revealed that the marine *Bp* group strains were grouped into three species: *B. pumilus*, *B. altitudinis* and *B. safensis*. All the three share a common ancestor. However, members of *B. altitudinis* were observed to cluster independently, separating from the other two, thus diverging from the others. Consistent with the universal nature of genes involved in the functioning of the translational machinery, the genes related to translation were enriched in the core genome. Functional genomic analyses revealed that the marine-derived and the terrestrial strains showed differences in certain hypothetical proteins, transcriptional regulators, K^+^ transporter (TrK) and ABC transporters. However, species differences showed the precedence of environmental adaptation discrepancies. In each species, land specific genes were found with possible functions that likely facilitate survival in diverse terrestrial niches, while marine bacteria were enriched with genes of unknown functions and those related to transcription, phage defense, DNA recombination and repair.

**Conclusion:**

Our results indicated that the *Bp* isolates show distinct genomic features even as they share a common core. The marine and land isolates did not evolve independently; the transition between marine and non-marine habitats might have occurred multiple times. The lineage exhibited a priority effect over the niche in driving their dispersal. Certain intra-species niche specific genes could be related to a strains adaptation to its respective marine or terrestrial environment(s). In summary, this report describes the systematic evolution of 52 *Bp* group strains and will facilitate future studies toward understanding their ecological role and adaptation to marine and/or terrestrial environments.

## Introduction

*Bacillus* are Gram-positive, aerobic or facultative anaerobic, rod-shaped bacteria that can produce highly resistant dormant endospores in response to various environmental stresses (Nicholson et al., 2000; Ravel and Fraser, 2005; Gioia et al., 2007; Earl et al., 2008; Setlow, 2014; Checinska et al., 2015). Benefiting from versatile metabolic capabilities and the ability for spore dispersal, *Bacillus* is ubiquitous in various natural environments. The genus comprises more than 500 species recognized to date^1^. Based on 1,172 core proteins, they have been phylogenetically divided into eight clades, including the Cereus, Subtilis, Simplex, Firmus, Jeotgali, Niacini, Fastidiosus, Alcalophilus clades (Patel and Gupta, 2019). *Bacillus pumilus* and their relatives belong to the Subtilis clade (Priest et al., 1987; Bhandari et al., 2013; Fan et al., 2017; Patel and Gupta, 2019).

The *Bacillus pumilus* group, abbreviated as the *Bp* group in this report, is a large group of *Bacillus* composed of *B. pumilus, B. safensis, B. altitudinis, B. xiamenensis*, *B. zhangzhouensis*, and *B. australimaris* (Liu et al., 2016). The members of this group have attracted wide attention for their applications in agriculture, industry and medicine (Berrada et al., 2012; Khianngam et al., 2014; Kumar et al., 2014; Lateef et al., 2015). At present, most of these industrial *Bp* group bacteria have been isolated from terrestrial ecosystems. However, the less explored marine *Bacillus* are even more diverse (Liu et al., 2013, 2017). To date, over one thousand marine *Bacillus* strains have been deposited in our collections^2^, approximately one-fifth of which belong to the *Bp* clade. They are frequently isolated from marine environments ranging from coastal and pelagic water columns to deep sea sediments, and most of these isolates have been preliminarily identified as either one of the three species of *B. pumilus, B. safensis*, and *B. altitudinis* based on 16S rRNA gene analysis (unpublished data). Among our collections, approximately 66% of *Bp* bacteria were isolated from marine sediments, 26% from marine water and 8% from marine animals. Due to the survivability of spores of this group under harsh conditions, we are not certain whether they are indigenous to marine habitats. In a study involving the cultivation of marine *Bacillus, B. pumilus* was found to be the predominant species in the coastal environment of Cochin, India (Parvathi et al., 2009). However, little is known about the geographic distribution of this group in global oceans and its speciation in various marine natural habitats (Ettoumi et al., 2009; Connor et al., 2010).

16S rRNA gene sequencing is a powerful tool for elucidating the phylogenetic diversity of bacterial families (Fox et al., 1980). However, it may not be applicable for the determination of the relationship of some species (Fox et al., 1992) within the groups of certain genera, such as *Bacillus*. In studies by other groups, methods such as *gyrB* gene sequence phylogeny, and matrix-assisted laser desorption/ionization time-of-flight mass spectrometry (MALDI-TOFMS) protein profiling have been applied to differentiate *Bp* group species (Dickinson et al., 2004; La Duc et al., 2004; Satomi et al., 2006). Previously, we found that the strains of the *Bp* group were closely related to each other, sharing over 99.5% 16S rRNA sequence similarity (Liu et al., 2013). To understand the phylogeny and biogeography of bacteria within this group, we performed a multilocus sequence analysis (MLSA) of selected marine *Bp* isolates. We used seven concatenated housekeeping genes with the order of *gyrB-rpoB-pycA-pyrE-mutL-aroE-trpB* to construct a phylogenetic tree. The choice of the genes is based on earlier work (Liu et al., 2013). The results showed that the bacteria of this group could be divided into six clades, and the three large clades were represented by *B. altitudinis, B. safensis*, and *B. pumilus*, respectively. Furthermore, the *gyrB*-based phylogenetic analysis of 73 terrestrial and marine isolates clearly showed that marine bacteria tended to cluster together in the three large clades (Liu et al., 2013). Although the MLSA provided clues about the phylogenetic relationship of the *Bp* strains, it is inefficient for resolving the taxonomic status of the strains of this group, as the housekeeping genes that are used account for only 0.10.2% of the genome (Hall et al., 2010). Moreover, MSLA data is not sufficient to understand bacterial adaptation to different natural habitats. Genome-based analysis can provide essential insights into species differentiation and environmental adaptation, including correlations with significant events in the evolutionary history of microbial species (Yerrapragada et al., 2009; Patan et al., 2018).

In recent years, pan-genomics has been widely applied for the investigation of bacterial species diversity, evolution, adaptability, and population structure (Lawrence and Hendrickson, 2005; Kettler et al., 2007; Muzzi et al., 2007; Tettelin et al., 2008; Vernikos et al., 2015; Tiwary, 2020). The pan-genome includes the core genome of genes common to all strains of a species and the dispensable or accessory genome, which consists of genes present in at least one but not all strains of a species and strain-unique genes (Medini et al., 2005). The essence of a species in terms of its fundamental biological processes and traits derived from a common ancestor is linked to the core genome. However, genetic traits linked to variations in virulence, adaptation and antibiotic resistance are more often governed by the dispensable or accessory genome (Tettelin et al., 2008; Maistrenko et al., 2020; Naz et al., 2020). Accordingly, it is of interest to estimate the sizes of the pan-genome, core genome, and new genes of a given species as novel genomes are added and to further identify the relative contributions of dispensable genomes and their relationships with specific adaptation to environmental niches (Zhang et al., 2018). Pan-genome analysis has been widely used for studying species evolution and functional gene changes (Wu et al., 2020), including those of several *Bacillus* species, such as *B. amyloliquefaciens, B. anthracis, B. cereus, B. subtilis*, and *B. thuringiensis* (Fang et al., 2011; Bazinet, 2017; Kim et al., 2017; Chun et al., 2019), most of which are terrestrial strains. In a previous report, we gained new insights into the phylogeny and differentiation of the *B. cereus* group via whole-genome analysis (Liu et al., 2015).

This report aimed at understanding the levels of differences between marine *Bp* bacteria and their terrestrial counterparts. In order to understand the taxonomy, diversity and environmental adaptation of marine *Bp* bacteria, we selected and sequenced 16 representative strains from various marine environments, including deep sea, coastal and polar areas. We performed phylogenetic, pan-genomic analyses with 36 other genomes downloaded from the NCBI, which were isolated mainly from soil, plants, animals, food, etc. The results based on these 52 *Bp* genomes will help elucidate the taxonomy of these strains and gain insights into their evolution and environmental adaptation.

## Materials and Methods

### Bacterial Strain Genome Collection

Based on our previous MLSA results (Liu et al., 2013) on the marine *Bp* bacterial isolates and their positions on different evolutionary branches, we selected 16 marine *Bp* bacteria for genome sequencing to avoid the bias caused by strain selection. Genomic DNA was extracted using an SBS extraction kit (SBS Genetech Co., Ltd. Shanghai, China) according to the manufacturers instructions. In addition, 36 genomes of *Bp*-group strains with isolation environment records were downloaded from the NCBI ftp site (Bacteria and Bacteria_DRAFT parts in http://ftp.ncbi.nlm.nih.gov/genomes). These strains were isolated from different habitats, such as soils, plants, marine environments, food, insects, air, children, and feces. Since only one genome of each of *B. xiamenensis*, *B. zhangzhouensis* and *B. australimaris* was available in the database, they were excluded from this analysis. The accession numbers of the 52 included strains are listed in Table 1, and information related to the 20 marine isolated strains are listed in Supplementary Table 1.

**TABLE 1:** Genome information of *Bp*-group strains (*indicates strains that we isolated and sequenced).

**Strains name**	**Isolation source**	**Genome size (Mb)**	**GC (mol%)**	**Protein coding sequences**	**Accession numbers**
S70-5-12* (marine isolated)	Marine surface water	3.64	41.30	3,739	JAAWWG000000000
BS1 *(marine isolated)	Marine bottom water	3.67	41.20	3,770	JAAWWH000000000
C16B11* (marine isolated)	Marine bottom water	3.68	41.30	3,730	JAAWWI000000000
C101* (marine isolated)	Marine sediment	3.68	41.20	3,783	JAAWWJ000000000
A23-8*(marine isolated)	Marine sediment	3.84	41.20	3,921	JABBCY000000000
Mn12* (marine isolated)	Marine Sediment	3.68	41.30	3,784	JABBCZ000000000
B-388 (land isolated)	Moth	3.74	41.20	4,030	GCA_000789425.2
41KF2b^*T*^	Air	3.68	41.30	3,681	GCA_000691145.1
RIT380 (land isolated)	Plant	3.97	41.00	4,031	GCA_001029865.1
NH21E_2*(marine isolated)	Marine sediment	3.78	41.50	3,774	JAAZTR000000000
B204-B1-5*(marine isolated)	Marine sediment	3.67	41.50	3,671	JAAZTO000000000
D95* (marine isolated)	Marine sediment	3.71	41.60	3,718	JAAZTP000000000
15-B04 10-15-3* (marine isolated)	Marine sediment	3.78	41.30	3,788	JAAZTQ000000000
NP-4* (marine isolated)	Marine surface water	3.71	41.60	3,711	JAAZQF000000000
S9 (marine isolated)	Lagoon	3.67	41.60	3,605	GCA_001653905.1
VK (land isolated)	Plant	3.68	41.60	3,472	GCA_000444215.1
FO-36b^*T*^	Clean-room air	3.73	41.60	3,716	GCA_003097715.1
MROC1 (land isolated)	Feces	3.61	41.70	3,557	GCA_001677975.1
sxm20-2* (marine isolated)	Marine sediment	3.76	41.50	3,830	JABBDA000000000
J33-1* (marine isolated)	Marine sediment	3.68	41.30	3,593	JABBDB000000000
C2-2* (marine isolated)	Marine sediment	3.7	41.20	3,676	JABBDC000000000
s8-t8-L9 * (marine isolated)	Marine sediment	3.71	41.30	3,679	JABBDD000000000
DW2J2 * (marine isolated)	Marine White shrimp	3.71	41.30	3,678	JABBDE000000000
SF214 (marine isolated)	Sea water	3.63	41.70	3,464	GCA_001468935.1
Fairview (marine isolated)	Marine subsurface water	3.83	41.55	3,764	GCA_000604385.1
RI06-95 (marine isolated)	Marine estuary	3.64	41.60	3,585	GCA_001183525.1
DSM 27 ^*T*^(land isolated)	Soil	3.92	41.80	3,845	GCA_000172815.1
3-19 (land isolated)	Soil	3.57	41.80	3,468	GCA_000714495.2
7P (land isolated)	Soil	3.58	41.80	3,461	GCA_000691485.2
BA06 (land isolated)	Soil	3.75	41.40	3,732	GCA_000299555.1
MTCC B6033 (land isolated)	Soil	3.76	41.40	3,697	GCA_000590455.1
TUAT1 (land isolated)	Soil	3.72	41.40	3,688	GCA_001548215.1
GR-8 (land isolated)	Soil	3.67	41.40	3,664	GCA_001191605.1
S-1 (land isolated)	Soil	3.69	41.30	3,687	GCA_000225935.1
NMTD17 (land isolated)	Soil	3.68	41.70	3,636	GCA_002998735.1
GBSW19 (land isolated)	Soil	3.8	42.10	3,571	GCA_002998655.1
NJ-V2 (land isolated)	Soil	3.79	41.30	3,783	GCA_001431785.1
INR7 (land isolated)	Plant	3.68	41.30	3,663	GCA_000508145.1
SH-B11 (land isolated)	Plant	3.86	41.30	3,776	GCA_001578165.1
SH-B9 (land isolated)	Plant	3.79	41.60	3,823	GCA_001578205.1
LK21 (land isolated)	Plant	3.67	41.60	3,638	GCA_001038905.1
LK12 (land isolated)	Plant	3.67	41.60	3,641	GCA_001043695.1
W3 (land isolated)	Honey	3.75	41.40	3,746	GCA_000972685.1
ku-bf1 (land isolated)	Wood borer insect	3.75	42.20	3,510	GCA_001543165.1
B4133 (land isolated)	Cereals, breads, pasta, pastries	3.72	41.20	3,684	GCA_000828455.1
B4127 (land isolated)	Cereals, breads, pasta, pastries	3.89	41.20	3,867	GCA_000828345.1
Boon (land isolated)	Childs hand	3.67	41.50	3,558	GCA_001444515.1
CB01 (land isolated)	Feces from crow roost	3.82	41.50	3,770	GCA_001675655.1
SAFR-032	Clean-room airlock	3.7	41.30	3,561	GCA_000017885.4
TH007 (land isolated)	Soil	3.69	41.40	3,648	GCA_001444735.1
FJAT-21955 (land isolated)	Soil	3.79	41.20	3,735	GCA_001420655.1
Leaf49 (land isolated)	Plant	3.93	41.00	3,971	GCA_001426125.1

### Genome Sequencing and Annotation

The genome sequencing and assembly of the 16 marine *Bp*-group strains was conducted on the Illumina HiSeq2000 platform with a sequencing read length of 101 bp by Shanghai Majorbio Bio-pharm Technology Co., Ltd. (Shanghai, China). The high-quality reads were assembled with the SPAdes program (Bankevich et al., 2012). Gene prediction and annotation were done using the Prokka program (Seemann, 2014). The predicted genes were also annotated by performing a BLAST search against the COG database with a cutoff *E*-value of 1e-5. The assembled genomes and annotated proteins were deposited in the GenBank database. The accession numbers for the 52 strains are listed in Table 1.

### Phylogenetic Analyses

All protein sequences of the 52 *Bp*-group strains and the outgroup of *Bacillus subtilis subsp. subtilis* 6051-HGW (GenBank accession: CP003329.1) were subjected to ortholog prediction using OrthoMCL (Li et al., 2003), with an *E*-value of 1e-15, an identity cut-off of 50%, a match cut-off of 50%, and other default settings. Gene clusters that were shared among all strains and contained only single gene copies from each strain were referred to as single-copy core genes. Only single-copy orthologous proteins were chosen for phylogenetic analyses. The single-copy orthologous proteins present in all 53 bacteria were aligned using MAFFT (Katoh and Standley, 2013). The alignment was trimmed using GBlock 0.91b (Castresana, 2000) and used to infer the evolutionary history of the strains with the Randomized Axelerated Maximum Likelihood algorithm (RAxML) and the JTTF model (Jones et al., 1992; Stamatakis, 2014). The reliability of the inferred tree was tested by bootstrapping with 1,000 replicates.

### Species Assignment Validation

Average nucleotide identity (ANI) values were calculated by using JSpecies software as previously described (Richter and Rossell-Mra, 2009). A Pearson correlation matrix was generated, and correlation analysis ordered by hierarchical clustering was performed following the procedures of Espariz et al. (2016). In addition, Mash software was chosen for clustering analysis (Ondov et al., 2016) and digital DDH values were determined using the Genome-to-Genome Distance Calculator (GGDC) web server^3^ using formula 2 as described by Auch et al. (2010) and Meier-Kolthoff et al. (2013).

### Pan-Genomics Analyses

All the genes from the 52 *Bp*-group strains were delineated into clusters with putative shared homology via the MultiParanoid (MP) method as implemented in the pan-genome analysis pipeline (PGAP) with a 50% cut-off (Zhao et al., 2011). The pan-genome and core genome profiles were then built. Functional enrichment of orthologous clusters was performed via the PGAP with the parameter function and used for the classification of clusters of orthologous groups (COGs). Twenty genomes of marine isolated strains (including 16 genomes from our collections and four from NCBI) and 32 genomes of strains isolated from terrestrial samples were then utilized to obtain pan-genome profiles via the PGAP. Finally, based on the phylogenetic species assignment results, the gene datasets of each species were used to obtain pan-genome profiles via the PGAP. The significance between COGs was determined with the STAMP (Parks et al., 2014) software using Fishers exact test (*P* < 0.05).

### Identification of Marine Adaptation Genes and Intra-Species Differences in Genes Related to Niche

Since dispensable genes in genomes confer selective advantages such as adaptation to different niches and antibiotic resistance (Vernikos et al., 2015), we first extracted the dispensable genomes of the 52 *Bp* group strains and then used OrthoMCL to cluster orthologous genes shared by all 20 *Bp* group strains isolated from marine environments. The orthologous genes that have been reported to be related to marine adaptation were considered candidate genes (Penn and Jensen, 2012; Li et al., 2020). COG annotation of candidate genes was then performed through a BLAST search against the downloaded COG database. In addition, we also used R package for core COG hierarchical clustering of 52 *Bp* group strains. Together with the phylogenetic species assignment results, we compared the different genes in each species between the marine isolated strains and the terrestrial isolated strains. We considered the marine specific genes in each species to be those that were present in at least one-third of the marine strains and were missing in all the terrestrial strains; in each species, terrestrial-specific genes were those that were shared by at least one-third of the terrestrial strains and were missing in all the marine strains.

## Results and Discussion

### General Features of the *Bp* Group Genomes

The analysis of the general features of genomes of the 52 *Bp* strains showed that the G + C contents ranged from 41.0 to 42.2 mol% and that the genome size ranged from 3.57 to 3.97 Mb (Table 1). Each strain of the *Bp* group contained 3,705 genes on average, ranging from 3,404 to 4,040 genes per genome, 7683% of which were assigned biological functions. With respect to their genome sizes, land isolated strains ranged from 3.57 to 3.97 Mb and the marine isolated strains ranged from 3.63 to 3.84 Mb. With respect to their G + C content, the terrestrial strains ranged from 41.0 to 42.2 mol% and the marine strains from 41.2 to 41.7 mol%. Additionally, the terrestrial strains have 3,4614,031 coding sequences, and the marine strains have 3,4643,921 coding sequences. Since closely related organisms have similar G (+C contents (Ochman et al., 2005), the similar GC percentages observed for the strains are suggestive of their close relatedness.

### Phylogenetic Relationships of *Bp* Group Strains Based on Core Genes

**FIGURE 1:**
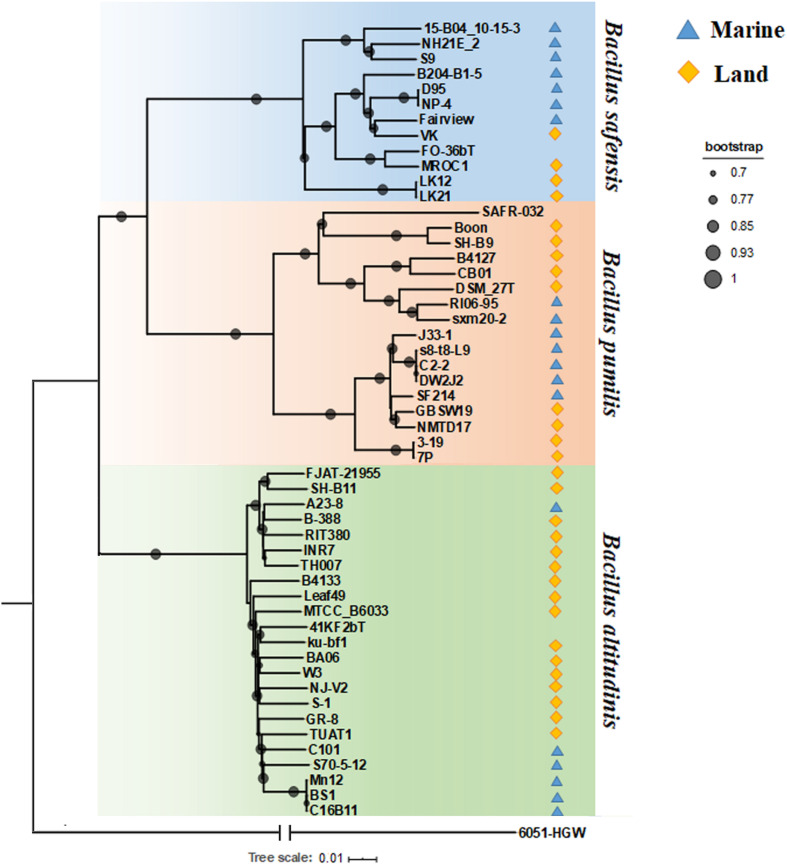
Phylogenetic tree based on total single-copy orthologous. Different colored shading represents the different isolation environments of the strains. Blue represents marine, and yellow represents land environment.

Since *Bp* group bacteria share high identities of the 16S rRNA gene (over 99.5%) (Liu et al., 2013), their phylogenetic relationships need to be resolved using robust higher resolution analyses. In order to do so, we combined core gene phylogenetic analysis, average nucleotide identity correlation and the Mash distance clustering method. *B. subtilis* subsp. *subtilis* 6051-HGW was selected as the out-group for the core genes in the phylogenetic analysis. First, 2,039 single copy orthologous proteins shared by 53 genomes, including strain 6051-HGW, were identified and then concatenated to construct an ML (maximum likelihood) phylogenetic tree (Figure 1). As shown in the core gene tree, these *Bp* strains were clearly clustered into three clades, corresponding to the three species represented by the type strains *B. pumilus* DSM 27^T^, *B. altitudinis* 41KF2b^T^, and *B. safensis* FO-36b^T^. Within each clade, it is our inference that the nomenclature given for the seven strains was incorrect or incomplete. Four of these strains, namely, TUAT1, MTCC B6033, SH-B11, and INR7, have been reported to cluster with other *B. altitudinis* strains in other studies (Espariz et al., 2016; Tirumalai et al., 2018). Our findings not only corroborate these previous results, but also place the three other strains, namely, TH007, Leaf49 and FJAT-21955, under *B. altitudinis*. We therefore recommend that all these seven strains be reassigned as *B. altitudinis.*

The ML tree based on 2,039 single-copy core genes showed that *B. pumilus* was more closely related to *B. safensis* than *B. altitudinis* (Figure 1), suggesting that *B. altitudinis* diverged from the other two. Furthermore, *B. altitudinis* included the majority of the *Bp* group strains, including six marine strains and 17 terrestrial strains. With the exception of strain A23-8, all of the marine strains of *B. altitudinis* were grouped together, suggesting that they shared a common ancestor (Figure 1). *B. safensis* included seven strains isolated from marine environments and five from land environments. Marine strains S9 (Ficarra et al., 2016), NH21E_2 and 15-B04 10-15-3 formed a monophyletic branch; the other branch of *B. safensis* contained strains from various environments, such as marine environments, plants, feces and air environments. The ancestor of the four marine-derived strains (D95, NP-4, Fairview, and B204-B1-5) might have been transmitted to the marine environment recently, since these strains were closely related to terrestrial strains within this subgroup. The *B. pumilus* subgroup, contained seven marine strains and ten terrestrial strains, which also clustered into two clades. However, despite occurring in the same subgroup, they differed in their isolation environments. For example, one mixed clade included both soil and marine isolates; four of the marine strains were clustered together, with the exception of SF214; the other clade contained strains of various origins, including samples from marine environments, soil, plants, children, air, food and crow feces, indicating that the bacteria in this clade were widespread in the environment. Overall, the marine bacteria from a given species tended to cluster together, while the terrestrial strains were more diverse and diverged more deeply at the tree nodes (Figure 1). Recently, the phylogeny of 81 bacteria of the genus *Bacillus* was examined using 196 core genes (Hernndez-Gonzlez et al., 2018). The authors found a clade consisting exclusively of species isolated from aquatic environments and considered some of the aquatic *Bacillus* species to share a single evolutionary origin. However, we found that the aquatic bacteria tended to group together only within species. We infer that for the *Bp* group strains, lineage takes priority over the niche effect in driving their divergence.

### Taxonomic Identity of *Bp* Strains Based on ANI, DDH, and Mash Distance

**FIGURE 2:**
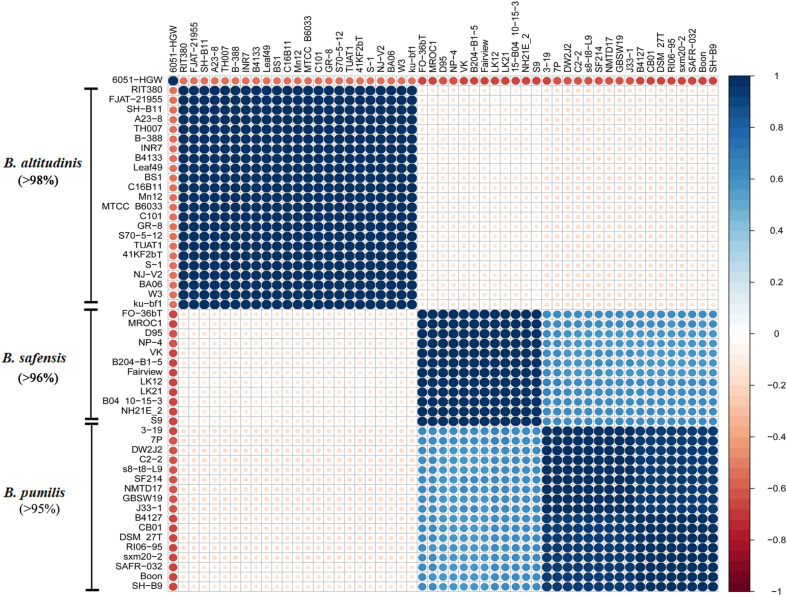
Correlation plot based on strains ANI values. ANI values between each strain were calculated using JSpecies software and employed for Pearson correlation matrix construction conducted using R. The plot shows the correlation constructed and ordered by hierarchical clustering using the R package corrplot. The minimum percentages of ANI values between the strains of a given cluster are indicated in brackets.

To confirm the taxonomic identity of these *Bp*-group strains, pairwise ANI values between the genomes of these strains were calculated, and correlation analysis was conducted to cluster related taxa (Figure 2 and Supplementary Table 2). The 52 strains clustered into three groups, which were identical to the species assignment results based on the core-gene phylogenetic tree. The largest group consisted of *B. altitudinis*, including 23 strains, sharing more than 98% ANI with each other and 8890% ANI with the other two species. Accordingly, *B. safensis* was composed of twelve strains, which shared more than 96% ANI with each other and 9093% ANI with *B. pumilus*. *B. pumilus* consisted of 17 strains, sharing more than 95% ANI with each other, similar to what was observed earlier (Satomi et al., 2006). The ANIs also suggested that *B. safensis* was more closely related to *B. pumilus* than *B. altitudinis*, corroborating previous observations (Tirumalai et al., 2018). In addition, a Mash-based tree was constructed for comparison with the above taxonomic results (Supplementary Figure 1). The Mash approach uses Mash distance to estimate the mutation rate between two sequences and to further cluster species (Ondov et al., 2016). The topology of the tree was similar to that of the core-gene tree in Figure 1 in terms of the species branches. Typically, two genomes belonging to the same species show more than 95% identity using ANI which corresponds to more than 6070% DDH values (Gupta and Sharma, 2015). When using 6070% DDH values as the standard cut-off range, the results from each of the following methods, viz., core genes tree, ANI, DDH and Mash distance tree, matched the other very well (Supplementary Table 3). If the cut-off DDH values were set at 70% for species assignment, the DDH results were similar to both the ANI results and the core genes phylogenetic tree, and showed that the *B. pumilus* strains formed two subclusters. These two subclusters strains shared 6070% DDH values.

With the combined application of multiple genomic-scale approaches, including core gene analysis, ANI correlation analysis, DDH values and Mash distance analysis, we were able to resolve the taxonomic status of both our marine and terrestrial *Bp* group strains, by assigning them into three species, and obtain a robust phylogenetic reconstruction. Thus, all these 52 strains were classified as either *B. pumilus*, or *B. altitudinis* or *B. safensis*. The *Bp* isolates show genomic features that are distinct from each other even as they share a common core, and a common ancestor.

### The Pan-Genome of the *Bp* Bacteria

The pan-genome is defined as the entire genomic repertoire of a given phylogenetic clade and encodes all possible lifestyles of the bacteria according to COG (Cluster of Orthologous Genes) analysis (Tettelin et al., 2008). The pan-genome is divided into three categories: the core genome, the dispensable genome and strain-unique genes. Here, (1) we explored the total pan-genome and functional content of *Bp* group strains; (2) we compared the pangenome of marine isolates with that of terrestrial isolates; and (3) we analyzed the pan-genome of three species according to the phylogenetic tree.

(1) Total Pan-genome Landscape

**FIGURE 3:**
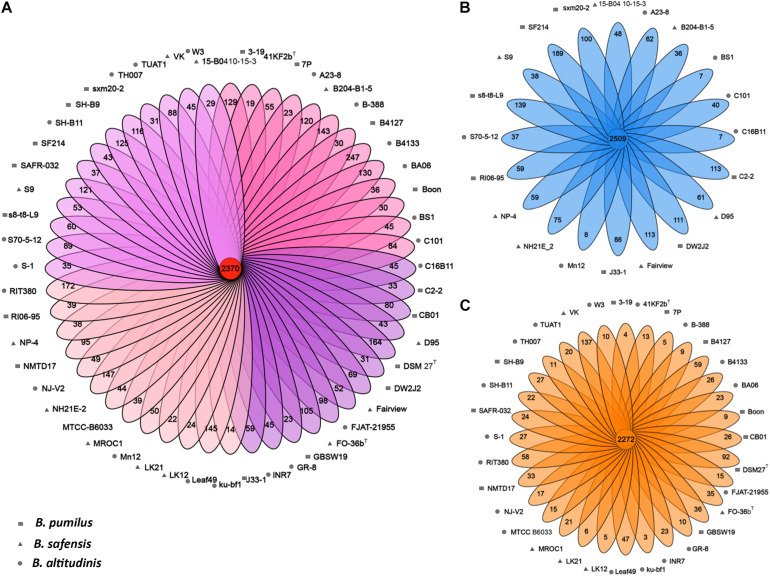
The pan-genomes of *Bp* group strains. **(A)** Flower plots showing the core gene number (in the center) and the strain-specific gene numbers (in the petals) in the 52 strains. **(B)** Flower plots showing the core gene number (in the center) and strain-specific gene number (in the petals) in the marine-isolated subgroups. **(C)** Flower plots showing the core gene number (in the center) and strain-specific gene number (in the petals) in the land-isolated subgroup.

The pan-genome of the 52 *Bp*-group strains contained 9,396 genes including 2,370 core genes (Figure 3A). For each strain, the proportion of the core genes was calculated, and the proportion ranged from 58.79 to 68.78%. In comparison, the proportion of the core genes corresponds to 74.01, 30.87, 26.01, and 38.75% of the total gene contents for the 52 *B. anthracis* strains, the 58 *B. cereus* strains, the 58 *B. subtilis* strains, and the 50 *B. thuringiensis* strains, respectively, as reported earlier (Kim et al., 2017). A total of 3,338 dispensable genes were found; dispensable genes were those that are present in two or more strains, not all strains (Medini et al., 2005; Tettelin et al., 2005; Vernikos et al., 2015), which account for 35.5% of the pan-genome. These dispensable genes are responsible for species diversity, environmental adaptation, and other characteristics of bacteria (Rouli et al., 2015). Strain-unique genes are those that are present in only one strain and are thought to be derived via horizontal gene transfer (Bentley, 2009); these genes accounted for 39.3% of the pan-genome genes among all tested *Bp* group bacteria, and the number of strain-unique genes varied from 14 to 247 between different strains. Both the dispensable genes and strain-unique genes constitute the non-core genes, representing subsets of the flexible genome. The *B. altitudinis* RIT380 strain possessed the highest proportion of non-core genes (41.21%), and strain *B. pumilus* 7P exhibited the lowest proportion (31.52%). This might reflect different levels of gene gains and losses during the evolution of these strains.

**FIGURE 4:**
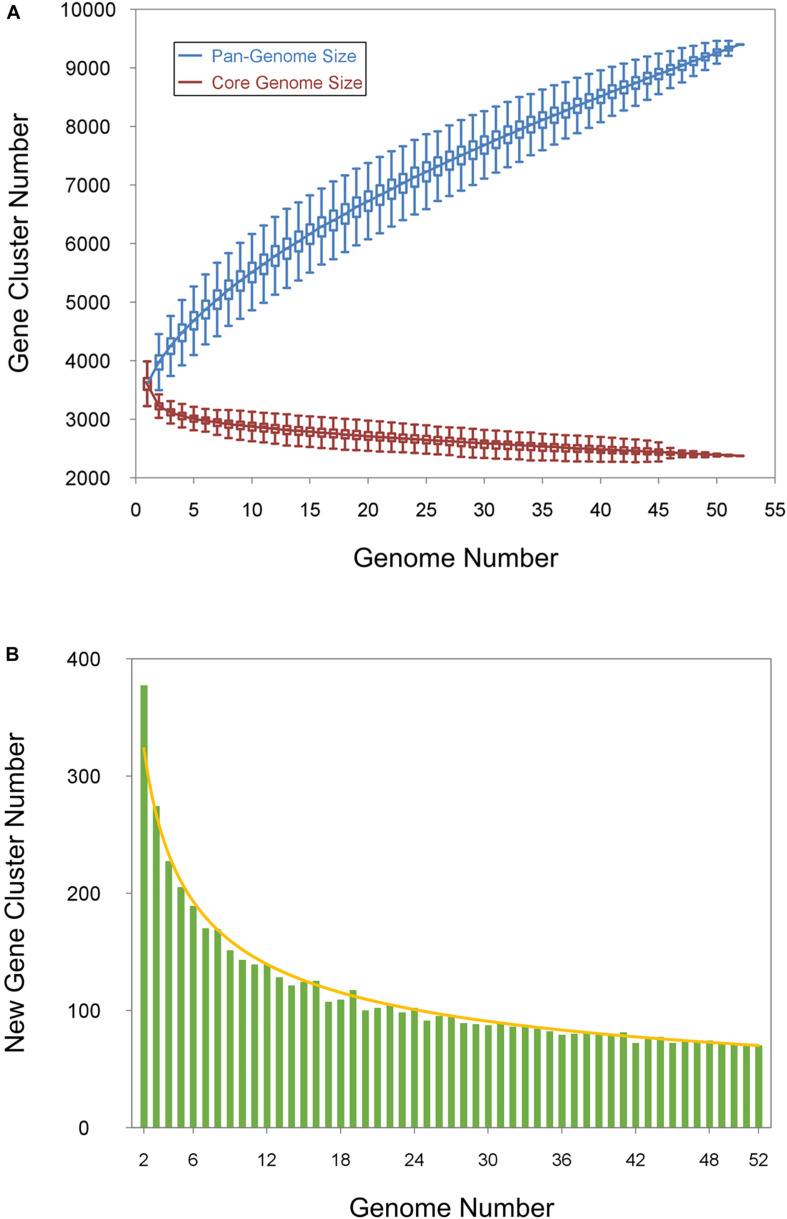
The pangenome curves of *Bp* group strains. **(A)** Core and pan-genome calculations for the *Bp* group. The blue curve shows an open pan-genome; the deduced pan-genome size is *y* = 726.2n^0.552^ + 2904.6 (*R*^2^ = 0.99998). The red curve represents the core genome, and the deduced core genome size is *c* = 939.4e^ 0.058n^ + 2379.1 (*R*^2^ = 0.94131). **(B)** New gene size and curve for *Bp*-group strains.

To understand the relationships between pan-genome size, the core gene number, and the strain numbers of the *Bp* bacteria, we plotted the fitted curves of the pan-genome profile of the 52 strains. To determine the core genome, the number of conserved genes found upon the sequential addition of each new genome was extrapolated by fitting a decaying function (Figure 4A, red curve), indicating that the average number of core genes converged to a relatively constant number. The core gene number in each genome varied slightly because of the involvement of duplicated genes and prologs in the shared clusters. As shown in Figure 4A, the blue curve increased with the addition of a new strain and was far from saturation, implying that the genetic repertoire of the species was still growing despite suitable adaptation to their diverse ecological niches. Thus, the pan-genome of the *Bp* group strains is open (Figure 4A, blue curve). This agrees with previous reports showing that environmental samples usually had open pan-genomes (Tettelin et al., 2005; Konstantinidis et al., 2009; Biller et al., 2015). In other words, the *Bp* group strains are expected to continue to gain genes and evolve in the future. The availability of a large genetic reservoir might be a key survival advantage for *Bp* group strains when facing environmental challenges. The number of new genes added by the additionally sequenced genomes was also plotted by fitting the decaying exponential in Figure 4B, and these new added genes determined the expansion of the *Bp* group pan-genome.

**FIGURE 5:**
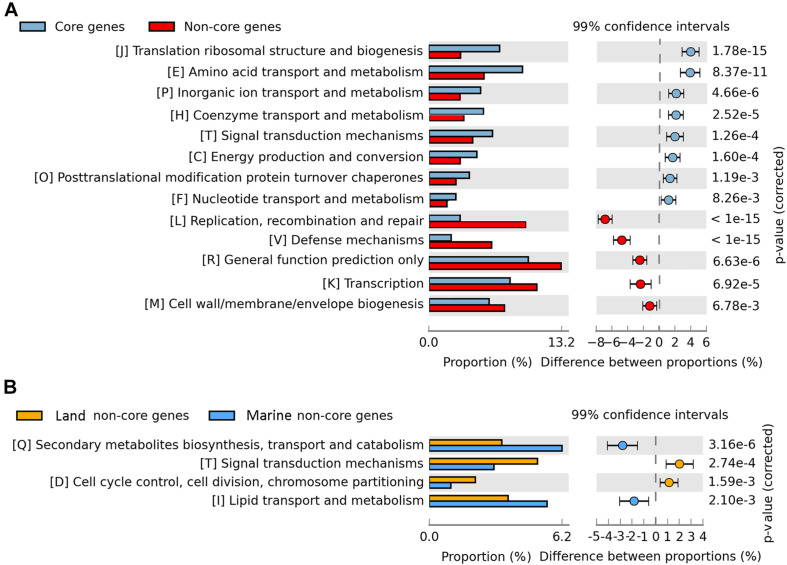
COG categories for total strains and marine vs. land strains. **(A)** COG categories of core genes and non-core genes. Blue represents core genes; red represents non-core genes. **(B)** Non-core gene COG categories of marine isolated strains and land isolated strains. Blue represents marine isolated strains; red represents land isolated strains.

To predict the functions of the genes that constitute the pan-genome, we took advantage of the COG functional classification. The COG category responsible for the highest proportion were poorly characterized genes (1021 genes), highlighting the important roles of these unknown genes corresponding to the undescribed physiology of *Bp* strains. The second most abundant category of genes in the pan-genome was general function predicted only including 989 genes and then by genes related to transcription (K) including 803 genes. The over representation of poorly characterized genes and general function predicted only in the COG categories is similar to what has been observed in other pan-genome studies (Sood et al., 2019; Gaba et al., 2020; Motyka-Pomagruk et al., 2020). Regulation at the transcriptional initiation level is probably the most common scenario for metabolic adaptation in bacteria (Leyn et al., 2013; Arrieta-Ortiz et al., 2015). The abundance of transcriptional regulators was consistent with the existence of complex transcriptional regulatory networks that support morphological and physiological differentiation. The most abundant core genes (40.41%) were associated with metabolism, whereas this category accounted for 26.57% of the strain-specific genes. In a given category of genes, the ratio of the percentage (of genes) in the core-genome, to its percentage in the non-core segment, showed that they could be arranged in a certain order of decreasing ratios. The genes involved in translation, ribosomal structure and biogenesis (J) had a ratio of 7.08:3.18%, while the other gene categories, namely, amino acid transport and metabolism (E), inorganic ion transport and metabolism (P), coenzyme transport and metabolism (H), had their core-genome/pan-genome composition percentages as 9.35/5.52%, 5.19/3.12%, and 5.44/3.47%, respectively. The translational machinery is widely accepted as a very ancient system and universal to life (Fox, 2010), and the translation machinery dominates the set of genes shared as orthologous across the tree of life (Bowman et al., 2020). Thus, given their universality, and essentiality, the genes involved in translation, ribosomal structure and biogenesis contribute to core genome stabilization. In the case of genes involved in repair (L) (3.11/9.69%), defense (V) (2.21/6.24%), transcription (K) (8.07/10.24%), and general function (R) (9.93/13.24%), the reverse was observed since they were better represented in the non-core segment than the core genome. This is suggestive of their possible roles in aiding the strains in their efficient response to different environments (Figure 5A).

(2) Pangenome of Marine and Terrestrial Groups

To detect the niche differentiation of *Bp* bacteria, they were divided into marine and land groups, and the pan-genome components were compared between marine and terrestrial strains. We found that the two groups exhibited a similar number of core genes, but the marine strains exhibited more strain-specific genes and fewer dispensable genes (Figures 3B,C), suggesting that horizontal gene transfer events have occurred more frequently among these marine strains. No significant difference was observed between the two groups in terms of COG category proportions in the core genome, suggesting that their core genes were highly functionally conserved. In the non-core genome, secondary metabolite biosynthesis, transport, and catabolism (Q) and lipid transport and metabolism (I) seemed to be over-represented in the marine group. The land group was enriched in signal transduction mechanisms (T), cell cycle control, cell division, chromosome partitioning (D) among the COG categories (Figure 5B). It has been suggested that land bacteria might respond quickly to environmental variation (Wan et al., 2019). At the same time, it has also been suggested that having dispensable genes involved in cell wall biogenesis, transport and metabolism of amino acids, carbohydrates, inorganic ions, and secondary metabolites, could be how certain members of *Azospirillum* adapt to terrestrial niches (Wisniewski-Dy et al., 2011). However, only twelve strains (refer to Table 1) covered in our study appear to be associated with hosts (plants and animals) on land. Our data is not conclusive enough to make a correlation between the non-core (dispensable) genes and the specific adaptations of the strains to their environment. Further studies are warranted to explore the same.

(3) Pangenomes of the Three Species

The pan-genome of each species included 6,306 orthologous genes in *B. altitudinis*, 6,284 in *B. pumilus*, and 5,262 in *B. safensis*. A total of 2,953 (46.8%), 2,693 (42.9%), and 2,924 (55.5%) core genes were identified in *B. altitudinis, B. pumilus* and *B. safensis*, respectively. The numbers of dispensable genes were 1,507 (23.9%), 1,728 (27.5%), and 1,187 (22.6%) in *B. altitudinis, B. pumilus* and *B. safensis*, respectively. A total of 1,846 (29.3%), 1,863 (29.6%), and 1,151 (21.9%) strain-unique genes were identified in *B. altitudinis, B. pumilus* and *B. safensis*, respectively (Table 2). COG analysis revealed that the core genes showed quite similar distributions in functional categories, reflecting their conservation in the three species. Among the non-core genes, *B. altitudinis* showed the enrichment of larger number of genes related to DNA recombination and repair (L) (5%) than the other two species (2% in *B. pumilus*, 1.8% in *B. safensis*). Such an enrichment could be enabling *B. altitudinis* strains to better cope with different stresses in land environments, in which they could be continuously exposed to DNA-damaging agents, including UVB, ozone, desiccation, rehydration, salinity, low and high temperature, and air and soil pollutants on land (Fang et al., 2017). *B. safensis* possessed more genes involved in carbohydrate transport and metabolism (G) (8% in *B. safensis*, 5.8% in *B. pumilus*, 5% in *B. altitudinis*), and secondary metabolite biosynthesis, transport and catabolism (Q) (4.8% in *B. safensis*, 2.2% in *B. pumilus*, 2% in *B. altitudinis*), than the other two species. Overall, *B. altitudinis* exhibited the largest pan-genome size, and *B. safensis* possessed the least variation in genetic diversity. *B. pumilus* exhibited the highest number of non-core genes and strain-specific genes, resulting in high genetic diversity in this species. This has likely facilitated the wide geographic distribution of *B. pumilus*.

**TABLE 2:** Pan-genome size of the three different species.

	***B. altitudinis***	***B. pumilus***	***B. safensis***
Core genes	2,953 (46.8%)	2,693 (42.9%)	2,924 (55.5%)
Dispensable genes	1,507 (23.9%)	1,728 (27.5%)	1,187 (22.6%)
Strain-specific genes	1,846 (29.3%)	1,863 (29.6%)	1,151 (21.9%)

### Marine Environmental Adaptations

In our dataset, 20 strains were isolated from marine environments, including 16 strains sequenced in this study and 4 genomes downloaded from NCBI. Since dispensable genome genes confer selective advantages such as adaptation to different niches and antibiotic resistance (Vernikos et al., 2015), we extracted the dispensable genes of the 52 *Bp* group strains and identified those shared by the 20 marine isolates. In the 20 marine bacterial isolates, 397 dispensable genes were found in total, including 41 hypothetical protein-coding genes. COG functional classification showed that 32 genes were transcription (K) related, which accounted for the highest proportion of the genes, such as transcriptional regulators belonging to the LysR family, AcrR family, GntR family, IclR family, IscR family, LacI/PurR family, MarR family, MerR family, MocR family, MurR/RpiR family, GlxA family, PucR family, etc. Regulatory proteins are thought to aid bacterial responses to specific environmental and cellular signals that modulate transcription, translation, or other events related to gene expression. Such adaptive responses facilitate bacterial survival in unstable environments (Romero-Rodrguez et al., 2015).

Generally, transcriptional regulators function via one- or two-component systems linking a specific type of environmental stimulus to a transcriptional response. The typical two-component systems usually consist of a membrane-bound sensor kinase (SK) and a DNA-binding protein known as a response regulator (RR) (Eswaramoorthy et al., 2010; Romero-Rodrguez et al., 2015). Of particular interest is the gene for the histidine kinase DesK. In *B. subtilis*, the histidine kinase DesK detects changes in temperature and activates the expression of a desaturase, thereby playing a role in increasing membrane fluidity and cold adaptation (Cybulski et al., 2010; Inda et al., 2014). All the 20 marine strains had the gene for DesK, whereas the homologs of DesK were found in only 7 land strains. The DesK homologs of the marine *Bp*-group strains showed 7080% similarity with *B. subtilis*. Since this gene is found in both marine and terrestrial strains, it is not clear if its presence confers any adaptive advantage to either the marine or terrestrial strains.

In addition, we found genes that could possibly play a role in coping with high salinity and oligotrophic conditions in marine bacteria. First, all the marine strains encoded glycine betaine transporters implying that they might use organic compatible solutes to maintain cellular osmotic balance. Glycine betaine is a well-known osmotic regulator that facilitates the accumulation of betaine, carnitine, and choline to maintain the cellular osmotic balance (Kempf and Bremer, 1998; Sleator and Hill, 2002). Compatible solutes such as glycine betaine generally stabilize proteins by preventing the unfolding and denaturation of proteins caused by heating, freezing, and dehydration to maintain cell volume and integrity in response to osmotic stress (Goh et al., 2011). Potassium (K^+^) ions are crucial for bacteria to regulate solute transport and adapt to changes in osmotic pressure. The K^+^ transporter (TrK) facilitates the accumulation of K^+^ in marine-derived strains, which is the primary strategy whereby many extremophiles survive in high-osmolality environments (Sleator and Hill, 2002; Roberts, 2004). There are various mechanisms for the accumulation of K^+^ in the cell, including TrK, Kdp, Kup and Ktr potassium transport systems (Yaakop et al., 2016). The Kdp system is considered a high-affinity, inducible system; the Ktr system requires Na^+^; the ability of the Kup system to transport potassium is weak; and the Trk system is a low-affinity but rapid potassium transporter (Cai and He, 2017). The presence of the gene encoding TrK in our marine strains is suggestive of a strategy to survive under relatively high osmolality, similar to what is observed in marine *Streptomyces* (Tian et al., 2016). Marine bacteria require Na^+^ for growth, and some use an Na^+^ circuit for various functions (Oh et al., 1991). Many Na^+^-dependent transporters were detected in our marine strains, including an Na^+^: H^+^ antiporter and Na^+^ symporters and transporters, which play essential roles in the pH and Na^+^ homeostasis of cells.

Another major aspect related to habitat adaptation is the availability of nutrients. Multiple sugar ABC transporters were identified in the marine strains, consistent with the report that there was an enrichment of ABC transporter genes in the genomes of deep-sea microorganisms (DeLong et al., 2006). The presence of multiple transporters could facilitate efficient uptake of nutrients under oligotrophic conditions, as reported for marine bacteria (Norris et al., 2020). The identification of sugar hydrolases, including glycosidases, beta-xylosidase, endoglucanase, xylanase/chitin deacetylase, and chitinase, implied that marine *Bp* group strains could sense and respond to sugar sources and degrade them as carbon and energy sources. Additionally, the existence of amino acid permeases, amino acid transporters, fructose permease, glucose uptake permease and phosphotransferase system proteins likely facilitate the absorbance of amino acids, oligopeptides, and carbohydrates by the marine strains. Most of these enzymes were also reported to enhance adaptation to marine oligotrophic environments in the marine bacterium *Zunongwangia profunda* SM-A87 (Qin et al., 2010). Drug/metabolite transporters (DMTs) and additional cation/multidrug efflux pump members are involved in drug/metabolite or homoserine lactone removal. Marine *Bp* group strains employ these transporters to defend against intracellular toxic substances, as a means of survival in the marine environment (Jack et al., 2001). One cation/multidrug efflux pump, one ABC-type dipeptide transport system, periplasmic component, two ABC-type sugar transport system, periplasmic component, one glycosyltransferase and one esterase/lipase COG were also recently detected among adaptive aquatic *Bacillus* COGs (Hernndez-Gonzlez et al., 2018), suggesting the existence of specific genes that play roles in niche adaptation.

### Intra-Species Specific Genes Indicating Speciation Between Marine and Terrestrial Strains

The marine isolates tended to cluster together in the core-gene phylogenetic tree, reflecting signals of differentiation and niche adaptation embedded in the genome. To make environmental groups more reasonable, COGs of core genes hierarchical clustering analysis for the 52 *Bp* group strains were performed (Supplementary Figure 2). The result was consistent with the core-gene phylogenetic tree which also exhibited marine origin strains gathered in each species. Therefore, to avoid discrepancies between different species, we compared marine and terrestrial strains within the same species. The presence/absence of genes as niche-specific genes and their COG distribution in the three species are summarized in Supplementary Table 4. Within each species, marine bacteria possessed more specific genes than land bacteria; however, more than half of these genes were of unknown functions.

Marine *B. pumilus* bacteria possessed more genes related to defense (V) and replication, recombination and repair (L) than those isolated from land (Supplementary Table 5). Additionally, we found that the genomes of our marine *B. pumilus* strains were enriched in phage and CRISPR elements (Supplementary Table 6), compared with land strains. *B. pumilus* phages have been isolated and characterized for their roles in horizontal gene transfer (Keggins et al., 1978; Lorenz et al., 2013; Badran et al., 2018), and implicated in acquisition of unique genes (Tirumalai et al., 2018) conferring traits such as spore radiation resistance (Tirumalai and Fox, 2013). Considering the large variety of phages in the marine environment (approximately 510 times higher than the number of bacteria), these genes might play roles in antiphage defense strategies (Wommack and Colwell, 2000; Doron et al., 2018). In comparison, land bacteria showed enrichment of genes specifically related to amino acid transport and metabolism (E) and mobile elements (X) and had different carbohydrate metabolism (G) related genes. Intriguingly, seven of the ten terrestrial strains contained a cyanide dehydratase that was not found in any marine *B. pumilus* strains. This cyanide dehydratase shared 73% amino acid identity with the cyanide dihydratase of *B. pumilus* C1, which has been proven to catalyze the conversion of cyanide to format and ammonia (Meyers et al., 1993). We could not explain why most of these land bacteria require the ability to perform cyanide detoxification. One possible clue toward answering this question is that cyanide-related compounds are common in terrestrial environments. A previous study indicated that cyanides are ubiquitously present in nature (Kumar et al., 2017), contributed by both anthropogenic activities and higher plant, fungal, and bacterial production (Baxter and Cummings, 2006; Larsen et al., 2004). Soil microbes can degrade cyanide to nitrites or form complexes with trace metals under aerobic conditions (Kjeldsen, 1999), suggesting that the land *B. pumilus* strains included in our study may utilize cyanide via cyanide dihydratase.

In the case of *B. safensis*, the marine strains had more specific genes related to transcription (K) and energy production and conversion (C), when compared with the land strains (Supplementary Table 5). The land strains have gained two multidrug transporters and specific genes in functional categories such as inorganic ion transport and metabolism (P), post-translational modification, protein turnover, and chaperones (O), compared with marine strain specific genes to cope with more diverse niches (Supplementary Table 5). All five terrestrial strains have gained the same ATP-binding protein of the ABC transporter, and two harbored the multidrug transporter EmrE, which was absent in the marine strains. These two specific proteins might help terrestrial strains to pump toxic substances outside the cell. Even within the same COG category, the proteins involved in amino acid and carbohydrate utilization were different between the land and marine strains. This reflected specialization in the metabolism of carbohydrates between land and marine strains, respectively. For example, the terrestrial strains have gained galactarate dehydrogenase and 5-dehydro-4-deoxyglucarate dehydratase; the former catalysis the transformation of galactarate to 5-dehydro-4-deoxy-D-glucarate, which is broken down by the latter enzyme (Deacon and Cooper, 1977).

In *B. altitudinis*, the marine strains contained specific genes involved in defense (V), replication, recombination and repair (L) against bacteriophages and other stresses, such as cold temperatures (Supplementary Table 5). The land strains were enriched in specific genes related to transcription (K), mobile elements (X), carbohydrate transport and metabolism (G), and some spore-related genes, such as the spore germination protein PD, the stage III sporulation protein AF, and the sporulation killing factor system radical SAM maturase. A previous study showed that 52 sporulation and germination genes were absent in the aquatic/halophile *Bacillus*, including the spore germination protein PD and the stage III sporulation protein AF (Alcaraz et al., 2010), which were also lost in our aquatic *B. altitudinis* strains. Overall, the large pangenome of *B. altitudinis* strains is an indicator of their versatility in terms of survival in diverse environments, such as soil, plants, food, feces, and animals.

## Conclusion

Our results of the genomic analysis of the *Bp* group indicate that the transition between marine and non-marine habitats has occurred multiple times during their evolutionary history. Both the land and marine *Bp* bacteria could be classified into three species. However, core gene based phylogenetic analysis showed that in each species, marine isolates tended to cluster together. The genomic plasticity of *B. pumilus* group members, and their potential for multi niche adaptation has been reported earlier (Branquinho et al., 2014). Thus, in each species, divergence was prone to occur with niche differentiation by gaining or losing different specific genes to adapt to different environments. With the generation of additional genomes of *Bp* group strains from a wider range of environments, insights into gene acquisition, deletion, and maintenance will facilitate a better understanding of the evolution of *Bp* group strains.

## Data Availability Statement

The datasets presented in this study can be found in online repositories. The names of the repository/repositories and accession number(s) can be found in the article.

## Author Contributions

ZS and XF planned and managed the projects. YL, QL, and LG conducted the experiments. XF and LG analyzed the data. XF and ZS interpreted the results and wrote the manuscript. All authors contributed to the article and approved the submitted version.

## Conflict of Interest

The authors declare that the research was conducted in the absence of any commercial or financial relationships that could be construed as a potential conflict of interest.

## Footnotes

1 http://www.bacterio.net/bacillus.html

2 www.mccc.org.cn

3 http://ggdc.dsmz.de/ggdc.php

## Supplementary Material

Supplementary Figure 1 Phylogenetic tree of *Bp* group strains generated with Mash software.

Supplementary Figure 2 Hierarchical clustering using core COGs.

Supplementary Table 1 Information of marine *Bp* group strains used for analyzing.

Supplementary Table 2 ANI values between each *Bp* group strain.

Supplementary Table 3 DDH values between each *Bp* group strain.

Supplementary Table 4 COG distribution of niche-specific genes in the three species.

Supplementary Table 5 Intra-species niche related specific genes of the three species.

Supplementary Table 6 *B. pumilus* strains information containing both phage and CRISPR.

Data Sheet 1 Alignments of protein (product) sequences of the core genes for phylogenetic tree construction.
