# Supplementary figures and images for: Bacillus pumilus Group Comparative Genomics: Toward Pangenome Features, Diversity, and Marine Environmental Adaptation
Source: Front Microbiol. 2021 May 7;12:571212. doi: 10.3389/fmicb.2021.571212 (PMC8139322; doi:10.3389/fmicb.2021.571212)

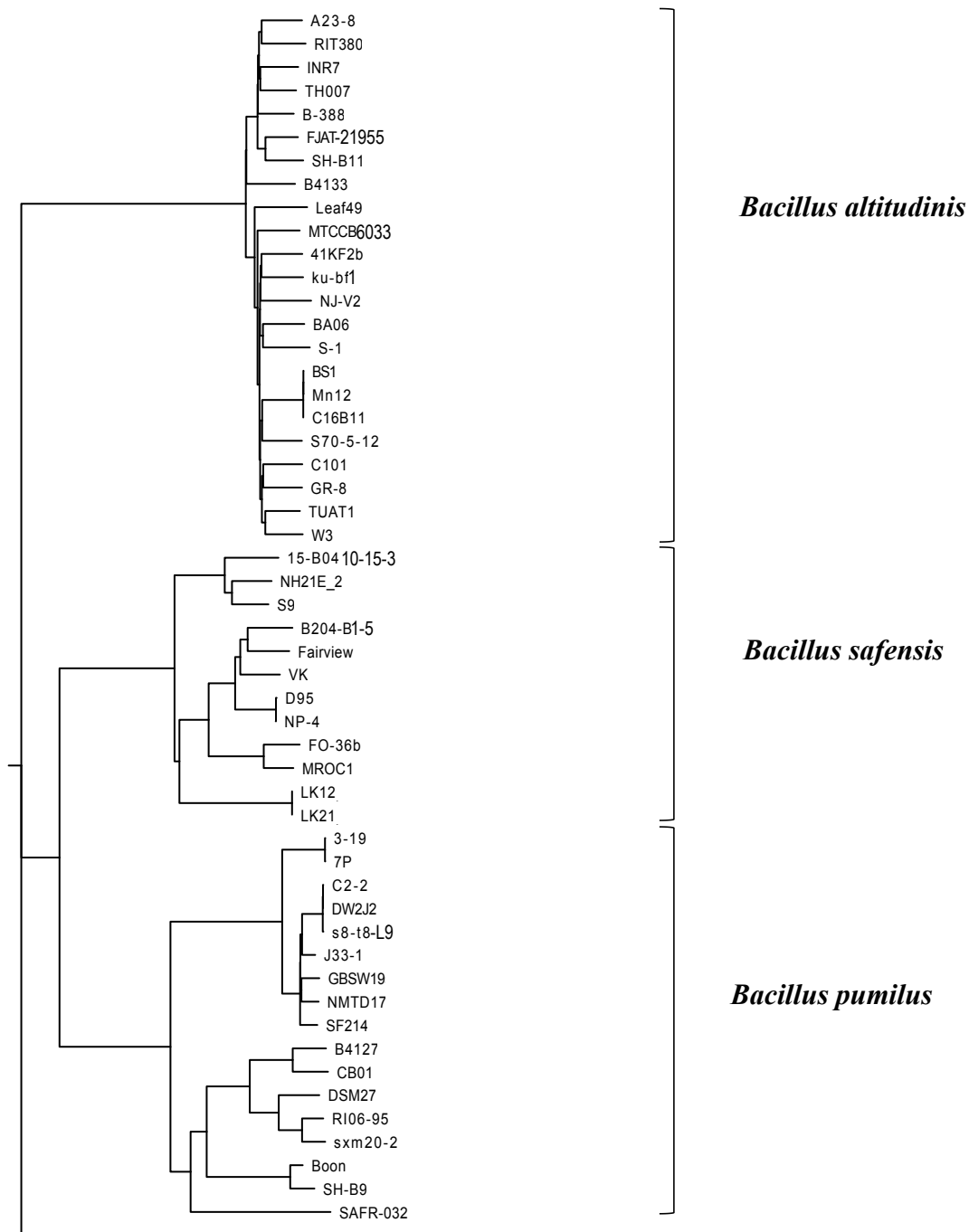

Supplement: Supplementary Figure 1 — Phylogenetic tree of Bp group strains generated with Mash software. [file Image_1.pdf]

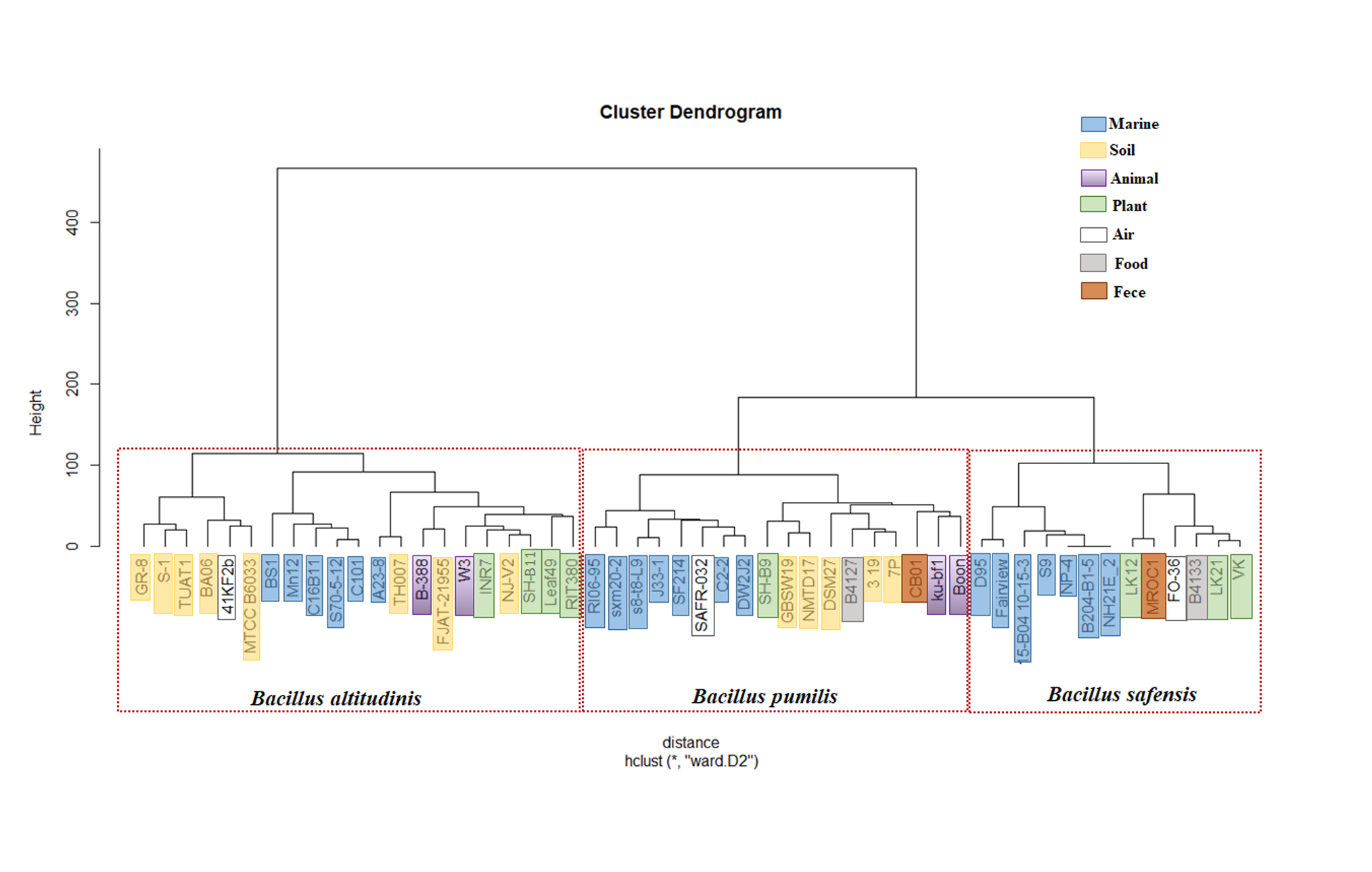

Supplement: Supplementary Figure 2 — Hierarchical clustering using core COGs. [file Image_2.tif]
